# *Notice to Readers*: Change in Continuing Education Activities for *MMWR* Weekly

**DOI:** 10.15585/mmwr.mm6704a7

**Published:** 2018-02-02

Effective February 1, 2018, *MMWR* Weekly will begin offering Continuing Education (CE) for one report per issue rather than the entire issue. The following types of CE will be available for each report: Continuing Medical Education (CME) for physicians, Continuing Nursing Education (CNE) for nurses, CE for certified health education specialists (CHES), and Continuing Education Units (CEU) for other health professionals. For reports relevant for veterinarians, American Association of Veterinary State Boards/Registry of Approved Continuing Education (AAVSB/RACE) will be available. CE is provided through CDC’s Training and Continuing Education Online (TCEO) system.

To obtain CE, users should log in to TCEO (http://www.cdc.gov/tceonline), search by the keyword “MMWR Weekly” to locate the appropriate issue, select the appropriate type of CE, complete the evaluation, and pass the posttest. CE can be obtained for 1 year from the date the activity is available in TCEO. No fee is charged for participating in these CE activities. Questions and comments about *MMWR* CEs can be submitted to mmwrq@cdc.gov.

